# Fatty Acid Composition in Various Types of Cardiac Adipose Tissues and Its Relation to the Fatty Acid Content of Atrial Tissue

**DOI:** 10.3390/nu10101506

**Published:** 2018-10-15

**Authors:** Katrin Hjelmgaard, Rikke B. Eschen, Erik B. Schmidt, Jan J. Andreasen, Søren Lundbye-Christensen

**Affiliations:** 1 Department of Cardiology, Aalborg University Hospital, 9000 Aalborg, Denmark; k.hjelmgaard@rn.dk (K.H.); r.bulow@rn.dk (R.B.E.); 2 Department of Clinical Medicine, Aalborg University, 9000 Aalborg, Denmark; jja@rn.dk (J.J.A.); solc@rn.dk (S.L.-C.); 3 Atrial Fibrillation Study Group, Aalborg University Hospital, 9000 Aalborg, Denmark; 4 Department of Cardiothoracic Surgery, Aalborg University Hospital, 9000 Aalborg, Denmark; 5 Unit of Clinical Biostatistics, Aalborg University, 9000 Aalborg, Denmark

**Keywords:** adipose tissue, diet, epicardial adipose tissue, pericardial adipose tissue, subcutaneous adipose tissue, site-specific differences, atrial tissue, fatty acids

## Abstract

Diet, with its content of various types of fatty acids (FAs), is of great importance for cellular function. Adipose tissue (AT) serves as a storage for dietary FAs, but after appropriate activation it may also offer important biological properties, e.g., by releasing adipokines and cytokines to the surrounding milieu. Such effects may depend on the diet and type of FA involved. Similarly, the composition of FAs in the heart is also likely to be important for cardiac function. We investigated samples of epicardial adipose tissue (EAT), pericardial adipose tissue (PAT), subcutaneous adipose tissue (SCAT), and tissue from the right atrial appendage to compare the FA compositions in patients undergoing elective cardiac surgery. Minor differences among AT compartments were found, while the comparison of atrial tissue and EAT showed major differences in saturated fatty acids (SFAs), monounsaturated fatty acids (MUFAs), and *n*-3 and *n*-6 polyunsaturated fatty acids (PUFAs). These findings may be of importance for understanding biological availability, dietary effects, and the effects of FAs on the heart.

## 1. Introduction

Epicardial adipose tissue (EAT) has been suggested to be involved in coronary artery disease and atrial fibrillation [1,2,3,4,5,6]. However, little is known about the composition of fatty acids (FAs) in EAT and how it interferes with closely related tissue sites (i.e., the coronary arteries and atrial tissue) and possibly affects cardiovascular disease (CVD). The composition of EAT is likely to be closely linked to the dietary intake of FAs but is also affected by their metabolism as well as the endogenous formation, particularly of saturated fatty acids (SFAs).

The adipose tissue (AT) composition of FAs is considered as a long-term biomarker reflecting dietary intake and the metabolism of FAs during the preceding one to three years [7,8,9,10]. Previously, FA composition of various AT compartments was assumed to be identical within an individual. However, more recent studies have reported differences in FA composition in AT from distinct regions [10,11,12,13]. This suggests that AT serves more complicated and specialized functions than was earlier believed, and that metabolic activities may differ between various AT compartments [10,14].

EAT has recently gained attention due to its possible effects on coronary artery disease and atrial fibrillation. The close anatomic relation of EAT to the adventitia of the coronary arteries and to the atrial tissue suggests that EAT has easy access and the potential to influence these heart structures through the release of specific FAs and their metabolic conversion products, e.g., eicosanoids, cytokines, and adipokines [3]. Notably, marine *n*-3 polyunsaturated fatty acids (PUFAs) are metabolized to less proinflammatory conversion products e.g., eicosanoids, rather than PUFA of the *n*-6 type. This suggests a relative anti-inflammatory effect of marine *n*-3 PUFA, which might be of clinical relevance as inflammation is believed to be of major importance for atherosclerosis and its clinical manifestations [15,16].

The aim of the study was to investigate potential differences between various AT compartments around the heart, e.g., EAT, pericardial adipose tissue (PAT), and subcutaneous adipose tissue (SCAT). Finally, we compared the FA composition of EAT to that of the atrium. These findings could potentially be of mechanistic relevance and have dietary implications.

## 2. Materials and Methods

### 2.1. Study Population

A total of 50 patients undergoing first-time elective cardiac surgery at Aalborg University Hospital, Denmark, were included in a previous study in which tissue samples were harvested, as described below, between 1 December 2014 and 30 April 2015 [17]. The original study was approved by the Research Ethical Committee of the Northern Denmark Region (N-20140070). Acceptance from the Danish Data Protection Agency was obtained in order to perform the present study (Study number: 2018-117). A total of 22 patients underwent isolated coronary artery bypass grafting (CABG), 14 underwent isolated valve surgery, 10 underwent combinations of CABG and valve surgery, while three underwent other forms of cardiac surgery. The characteristics of the total patient population are given in Table 1.

### 2.2. Atrial and Adipose Tissue Samples

Tissue samples were obtained during surgery. A right atrial tissue sample from the atrial appendage was collected from each patient by cutting the top of the atrial appendix at the canulation site when the venous cannula for the heart-lung machine was inserted. After SCAT (max 1 cm^3^) was obtained from above the sternum, and PAT (max 1 cm^3^) was obtained from below the sternum, EAT was obtained from above the right ventricle.

The samples were transported to the research laboratory and cleaned immediately. The atrial tissue samples, 20–30 mg, were mixed with 1.0 mL methanol (MeOH) containing butylated hydroxytoluene (BHT) (100 µg/mL) and 2.0 mL chloroform (CHCL_3_). The suspension was furthermore sonicated for 2 min to rupture the cells, vials were filled with nitrogen, and the sample was stored at −20 °C until further analysis. The AT samples were cut into pieces, transferred into cryo vials, overlaid with nitrogen, and frozen at −80 °C until analysis.

### 2.3. Preparation of Atrial Tissue Samples

The extraction of the total lipids from the atrial tissue was carried out using a modified version of a method described by Folch et al. [18]. The samples were thawed, 750 µL NaCl added, mixed, centrifuged, and the organic phase was collected. The extraction was repeated with 1.0 MeOH containing BHT, 2 mL CHCL_3_ and 750 µL NaCl mixed, and centrifuged. The organic phase was collected, and the combined organic phase was dried under nitrogen for 45 min at 30 °C, dissolved in 1 mL chloroform, and briefly mixed.

The separation of the phospholipid FA fraction from the total lipids from atrial tissue was conducted according to a modified version of the method described by Burdge et al. [19]. The total lipid fraction dissolved in 1 mL chloroform was transferred to a Bond Elut NH_2_ column of 200 mg (Agilent Technologies, Santa Clara, CA, USA), preconditioned with 4 mL hexane, and washed with 4 mL of chloroform afterwards. The phospholipid FA fraction was eluted with 2 mL chloroform-methanol 3:2, followed by 2 mL of methanol, and afterwards the tubes were dried under nitrogen for 1 h at 40 °C.

For the methylation of the FAs from atrial tissue, tubes were incubated at 50 °C for 5 min before the FAs were dissolved in 250 µL of warm heptane (50 °C), briefly mixed, and 12.5 µL of 2 M potassium hydroxide dissolved in methanol was added to the mixture according to the method described by Bannon et al. [20]. The tubes were briefly mixed a second time and methylated for 2 min at 50 °C, mixed twice for 1 min, and then stored at room temperature for 10 min, packed with aluminum foil. Subsequently, the tubes were centrifuged at 3220 g for 10 min at 10 °C, and the supernatant was transferred into vials suitable for gas chromatography.

### 2.4. Preparation of Adipose Tissue Samples

Due to its content of pure triglycerides, AT does not require extraction or separation before further handling. Before analysis, the biopsies were thawed, and 1–2 mg was used for methylation.

Methylation of the FAs from AT was performed according to the same method described for atrial tissue. Tubes were incubated at 50 °C for 5 min before the FAs were dissolved in 250 µL of warm heptane (50 °C), briefly mixed, and 12.5 µL of 2 M potassium hydroxide dissolved in methanol was added to the mixture according to the method described by Bannon et al. [20]. The tubes were briefly mixed a second time and methylated for 2 min at 50 °C, mixed twice for 1 min, and then stored at room temperature for 10 min, packed with aluminum foil. Subsequently, the tubes were centrifuged at 3.220× *g* for 10 min at 10 °C, and the supernatant was transferred into vials suitable for gas chromatography analysis.

### 2.5. Analysis of Fatty Acid Composition in Adipose Tissue and Atrial Tissue

This has been described in detail previously [17]. Briefly, the FA composition was analyzed through gas chromatography using a Varian 3900 GC with a CP-8400 auto sampler (Varian, Middleburg, The Netherlands) equipped with a flame ionization detector. In split injection mode, a CP-sil 88 for FAME, 60 m × 0.25 mm ID capillary column (Agilent Technologies, Santa Clara, CA, USA), with temperature programming from 90° to 210 °C and steady flow, was used. Helium was utilized as the carrier gas. The individual FAs were identified by their relative retention time using commercially available standards (Nu-chek-Prep, Inc., Elysian, MN, USA).

The results were not quantified according to the internal standards, but were obtained from a chromatogram, expressing the results of the individual FAs (*n* = 34) as a percentage of the total FAs.

### 2.6. Statistical Analysis

The distribution of the baseline characteristics and FA composition in the different compartments was described by means and standard deviations (SD) for continuous variables, and by number and proportion (%) for discrete variables. Differences between the compartments were expressed by mean difference and a 95% confidence interval (CI). Pearson correlations between FAs in atrial tissue and AT compartments were calculated for all the FAs. Correlations were illustrated using scatterplots for alpha-linoleic acid (ALA), eicosapentaenoic acid (EPA), docosahexaenoic acid (DHA), linoleic acid (LA), and arachidonic acid (AA) with regression lines and confidence bands superimposed between atrial tissue and the different AT compartments.

Comparisons between compartments of FAs were performed using repeated measures ANOVA (Analysis of Variance). The calculation of CIs was done using bootstrap with 5000 replications due to the heterogeneity of the variances and the potential departures from the normal distribution.

A *p* value < 0.05 (two-tailed) was considered as statistically significant. Analyses were performed using STATA statistical software version 15.1 (StataCorp LLC, College Station, TX, USA).

## 3. Results

The atrial tissue sample from one patient was insufficient for FA analysis and this patient was excluded, leaving 49 patients for the final analyses.

Table 2 shows the FA composition of EAT, PAT, SCAT, and atrial tissue. Oleic acid was the major FA in all the ATs, followed by palmitic acid and linoleic acid. Palmitic acid was the major FA in atrial tissue, followed by oleic acid and arachidonic acid (AA). Between atrial tissue and AT, major differences were seen in all FA groups (SFAs, monounsaturated fatty acids (MUFAs), and *n*-3 and *n*-6 PUFAs) with major differences observed in particular for oleic acid, AA, and palmitic acid.

Comparisons between EAT, PAT, and SCAT are given in Table 3. Small but statistically significant differences were found in all AT compartments, reflected by the Global Test column in Table 3. The content of EPA was statistically significantly higher in EAT, when compared to PAT and SCAT, whereas ALA was significantly lower in EAT when compared to PAT and SCAT. Concerning *n*-6 PUFA, EAT contained significantly higher amounts of AA compared to PAT and SCAT.

Differences between the FA composition of atrial tissue and EAT are shown in Table 4. Statistically significant differences were found for all FAs investigated. The content of *n*-6 PUFA was statistically significantly higher in atrial tissue when compared to EAT, and the difference was mainly driven by the content of AA. The content of *n*-3 PUFA was statistically significantly higher in atrial tissue compared to EAT, which was mainly driven by the high amounts of DHA in atrial tissue. The content of ALA was significantly lower in atrial tissue compared to EAT.

**Figure 1:**
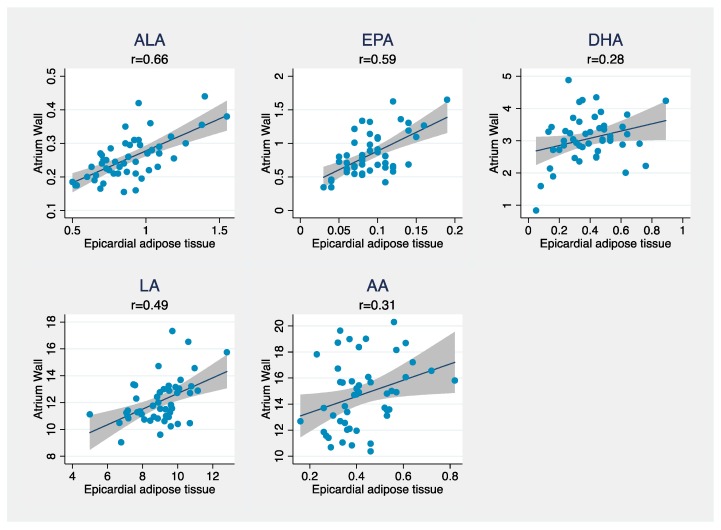
Scatterplots showing the relation between epicardial adipose tissue and atrial tissue for ALA, EPA, DHA, LA, and AA. Pearson’s correlation coefficient is denoted by *r*. ALA, alpha-linoleic acid; EPA, eicosapentaenoic acid; DHA, docosahexaenoic acid; LA, linoleic acid; AA, arachidonic acid.

Correlations between the major PUFAs in atrial tissue and EAT are given in Figure 1 and demonstrated in scatterplots. The highest correlation coefficients were found for ALA (*r* = 0.66), EPA (*r* = 0.59), and LA (*r* = 0.49).

**Figure 2:**
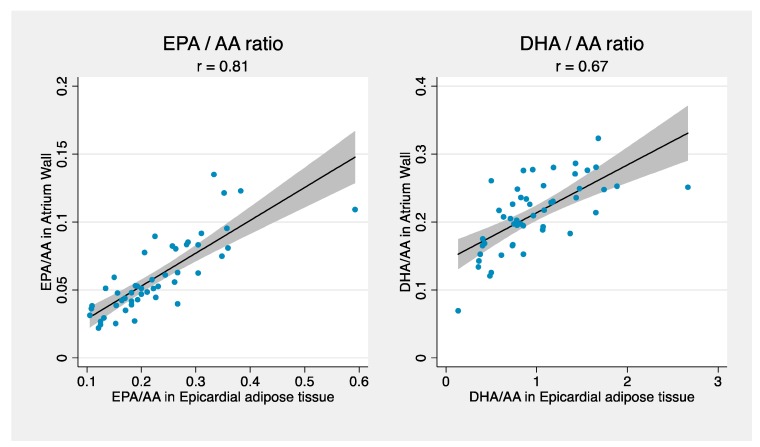
Scatterplots showing the relation between EPA and AA in epicardial adipose tissue and atrial tissue and DHA and AA in epicardial adipose tissue and atrial tissue. Pearson’s correlation coefficient is denoted by *r*. EPA, eicosapentaenoic acid; DHA, docosahexaenoic acid; AA, arachidonic acid.

Correlations between the ratios of EPA/AA and DHA/AA, respectively, in atrium and EAT, are shown in the scatterplots in Figure 2. They demonstrate high correlations for both ratios, in particular for EPA/AA.

## 4. Discussion

The present study showed that there were minor but overall statistically significant differences in the FA composition between the different AT compartments. Furthermore, there were major differences between the FA composition of EAT and atrial tissue in all FA groups. Atrial tissue had more SFAs than AT, and contained more *n*-3 and *n*-6 PUFA compared to EAT. However, EAT contained more MUFA. Among the AT compartments, small but statistically significantly differences were found, and EAT contained more EPA and AA compared to PAT and SCAT. Correlation coefficients comparing EAT and atrial tissue showed high correlations between ALA, EPA, LA, and the ratios EPA/AA and DHA/AA. The significant difference between the content of various FAs in adipose and atrial tissue is puzzling and, apparently, these tissues handle FAs differently. The high content of *n*-6 PUFAs and long-chain marine *n*-3 PUFAs in the atrium—increasing the susceptibility for lipid peroxidation—may suggest important functions for these FAs in relation to atrial function.

The study had some strengths: To our knowledge, this study was the first to investigate and compare the FA composition in EAT, PAT, and SCAT, and, moreover, to compare EAT with atrial tissue. The FA composition was very different in atrial tissue compared to EAT, and it was therefore a strength that a bootstrap analysis with 5000 replications was carried out, which validated our results. The biopsies were taken from each patient on the same day and from comparable sites, which may also be considered as a strength.

The study also had some limitations: The sample size was rather low, which decreased our statistical power and increased the risk of type II errors. The findings in the present study are limited to patients with valve or coronary artery disease undergoing elective cardiac surgery, which may not be representative of the general population. Also, due to the low numbers of patients, we were unable to provide a meaningful analysis separately for patients with coronary artery disease and valve disease and separate analyses in patients with various diseases such as hypertension and diabetes. The AT content of individual FAs was reported as percentages of total FAs, meaning that the results depended on the content of the other FAs, which could have skewed our data. It is also a weakness that we did not obtain AT from the buttocks for direct comparison with the other AT compartments, because this AT site might have other metabolic properties and is a tissue site often used in other studies. Importantly, since this study was a descriptive cross-sectional study, it could not establish causality; it could only describe and imply associations. Finally, an important limitation was the lack of dietary data from the patients. It would have been of interest to compare the dietary intakes of various types of FAs with the composition of AT and atrial tissue. It should, however, be kept in mind that AT reflects the long-term intake of FAs, which might be difficult to directly relate to recent diets.

The FA composition in AT has been examined by many other studies; however, only a few studies have investigated the site-specific differences between different AT compartments [10], and to our knowledge no studies have focused on ATs closely related to the heart. However, studies that have investigated these differences, mainly between two subcutaneous sites or compared subcutaneous sites with intra-abdominal AT, have reported significant differences in FA composition and metabolism [11,13,21]. EAT has been suggested to be a particularly metabolic active tissue that might function as a lipid-storing endocrine organ that secretes cytokines and chemokines [2,3,22,23]. However, only a few studies have in fact studied the FA composition of EAT. A study performed by Pezeshkian et al. investigated the FA compositions of EAT and SCAT taken from the leg in 42 patients with coronary artery disease [24] and reported similar results to our study, and in addition found EAT to contain more SFAs compared to SCAT. However, our study differed regarding the other FAs, as we found small but statistically significant differences between several FAs. Pezeshkian et al. only studied patients with coronary artery disease [24], while we investigated a group of patients with valve or coronary artery disease. Coronary artery disease is a condition known to be associated with a more biologically active EAT [25,26], and could therefore have had an effect regarding the differences between the results of the studies. Thus, the small but significant differences in FA composition between EAT, PAT, and SCAT confirm that metabolic activities, e.g., deposition and utilization of FAs, may differ among AT compartments.

The EAT has been associated in multiple recent studies with CVD such as coronary artery disease [27,28], cardiac hypertrophy [6], and atrial fibrillation [4,6]. To our knowledge, the present study is the first to investigate and compare the FA composition in EAT and atrial tissue. The lack of a fibrous fascia between the tissues may facilitate the abundance of adipocytes or the diffusion of adipokines or free FAs released from EAT into the adventitia of the coronary arteries or the myocardium [3,27]. A recent study performed by Venteclef et al. showed that secretomes from EAT did indeed promote myocardial fibrosis through the release of adipo-fibrokines [6], confirming the interaction between these two tissues. Dietary *n*-3 PUFAs, in particular EPA and DHA, are believed to be metabolized to compounds that possess anti-inflammatory and antiarrhythmic effects, among others [16,29,30], compared to the more common *n*-6 PUFAs. Therefore, a change in FA composition of EAT by dietary changes (e.g., increased intake of marine *n*-3 PUFAs) might affect the secretion of metabolically active substances from EAT and thereby potentially reduce coronary atherosclerosis. A possible effect of *n*-3 PUFA on arrhythmias would likely involve the incorporation of *n*-3 PUFAs into the atrium, and therefore the content of types of FA in the atrium is likely to be of importance, although circulating *n*-3 PUFA in plasma cannot be excluded from having an effect on arrhythmias as well [17]. The markedly higher content of marine *n*-3 PUFA, in particular DHA (but not of ALA), in atrial tissue than in AT might represent a mechanism for the body to reduce arrhythmias, perhaps through an increased conversion of ALA to long-chained *n*-3 PUFAs. This is hypothetical but could have important dietary implications. Further research is warranted to elucidate the interaction between the FA composition of EAT and atrial tissue and the clinical importance of these findings, including the significance of the apparent high levels of long-chain *n*-3 PUFAs in atrial tissue.

In summary, we found small but significant differences in the FA composition between EAT, PAT, and SCAT. Major differences were found between EAT and atrial tissue and a high correlation, especially for EPA, was found as well as for the EPA/AA ratio. Further investigations regarding EAT are recommended to elucidate the mechanisms of this tissue and how it interacts with coronary arteries, atrial tissue, and surroundings. Furthermore, the high levels of long-chain *n*-3 PUFAs in atrial tissue and its significance for arrhythmic risk require further exploration as this could have important implications for dietary recommendations for the prevention of heart disease.

## Figures and Tables

**Table 1:** Baseline characteristics of patients.

Variables	*n* = 49
Age, years	66.0 (10.4)
Men:Women	39:10
Weight, kg	81.1 (13.6)
BMI, kg/m^2^	26.9 (3.7)
Current smoking	32 (65.3%)
Prior MI	9 (18.37%)
Hypertension	34 (69.4%)
Diabetes mellitus	12 (24.5%)

Data are presented as mean (Standard Deviation (SD)) or number of participants (%). BMI, body mass index; MI, myocardial infarction.

**Table 2:** Fatty acid composition (% of total FA) in epicardial adipose tissue, (EAT), pericardial adipose tissue (PAT), subcutaneous adipose tissue (SCAT), and atrial tissue.

	EAT	PAT	SCAT	Atrium
Total SFA	31.05 (2.64)	30.84 (2.55)	30.18 (2.60)	43.61 (2.22)
14:0 (myristic acid)	2.58 (0.52)	2.83 (0.56)	2.66 (0.53)	0.99 (0.22)
16:0 (palmitic acid)	23.31 (1.92)	22.62 (2.07)	22.68 (2.08)	34.35 (2.05)
18:0 (stearic acid)	5.17 (1.17)	5.39 (0.97)	4.84 (0.93)	8.27 (0.70)
Total MUFA	45.70 (2.27)	46.36 (2.23)	46.96 (2.27)	16.09 (1.82)
18:1 *n*-9 (oleic acid)	45.57 (2.27)	46.24 (2.24)	46.85 (2.27)	16.04 (1.81)
Total *n*-3 PUFA	1.74 (0.38)	1.59 (0.34)	1.56 (0.31)	5.40 (0.99)
18:3 *n*-3 (ALA)	0.89 (0.23)	0.97 (0.26)	0.94 (0.23)	0.26 (0.07)
20:5 *n*-3 (EPA)	0.09 (0.03)	0.08 (0.03)	0.08 (0.03)	0.84 (0.32)
22:5 *n*-3 (DPA)	0.37 (0.14)	0.29 (0.13)	0.31 (0.14)	1.24 (0.24)
22:6 *n*-3 (DHA)	0.39 (0.18)	0.26 (0.12)	0.23 (0.11)	3.07 (0.73)
Total *n*-6 PUFA	9.38 (1.43)	9.58 (1.51)	9.79 (1.47)	26.80 (2.90)
18:2 *n*-6 (LA)	8.96 (1.41)	9.30 (1.51)	9.44 (1.45)	12.07 (1.68)
20:4 *n*-6 (AA)	0.42 (0.13)	0.28 (0.09)	0.34 (0.13)	14.73 (2.64)

Results are given as mean (SD) (% of total fatty acids). SFA, saturated fatty acid; MUFA, monounsaturated fatty acid; PUFA, polyunsaturated fatty acid; ALA, alpha-linoleic acid; EPA, eicosapentaenoic acid; DPA, docosapentaenoic acid; DHA, docosahexaenoic acid; LA, linoleic acid; AA, arachidonic acid.

**Table 3:** Regional differences between the three adipose tissue compartments.

	PAT vs. SCAT	EAT vs. SCAT	EAT vs. PAT	Global Test
Total *n*-3 PUFA	0.05 (0.02–0.08)	0.12 (0.08–0.16)	0.07 (0.02–0.12)	*p* < 0.001
18:3 *n*-3 (ALA)	0.03 (0.01–0.05)	−0.05 (−0.08–−0.02)	−0.08 (−0.11–− 0.05)	*p* < 0.001
20:5 *n*-3 (EPA)	−0.01 (−0.01–−0.00)	0.01 (0.00–0.01)	0.01 (0.01–0.02)	*p* < 0.001
22:6 *n*-3 (DHA)	0.03 (0.01–0.05)	0.16 (0.13–0.19)	0.13 (0.10–0.17)	*p* < 0.001
Total *n*-6 PUFA	−0.21 (−0.29–−0.13)	−0.41 (−0.54–−0.27)	−0.20 (−0.34–−0.05)	*p* < 0.001
18:2 *n*-6 (LA)	−0.15 (−0.22–−0.08)	−0.49 (−0.63–−0.35)	−0.34 (−0.49–−0.19)	*p* < 0.001
20:4 *n*-6 (AA)	−0.06 (−0.09–−0.04)	0.08 (0.06–0.11)	0.15 (0.12–0.18)	*p* < 0.001

Results are given as mean differences (%) (CI). The rightmost column contains the *p* value for the global hypothesis that the content of FAs did not differ between the AT compartments. PAT, pericardial adipose tissue; SCAT, subcutaneous adipose tissue; EAT, epicardial adipose tissue; PUFA, polyunsaturated fatty acid; ALA, alpha-linoleic acid; EPA, eicosapentaenoic acid; DHA, docosahexaenoic acid; LA, linoleic acid; AA, arachidonic acid.

**Table 4:** Differences between atrial tissue and epicardial adipose tissue (EAT).

	Atrium vs. EAT
Total *n*-3 PUFA	2.79 (2.54–3.04)
18:3 *n*-3 (ALA)	−0.63 (−0.68–0.58)
20:5 *n*-3 (EPA)	0.74 (0.66–0.83)
22:6 *n*-3 (DHA)	2.68 (2.48–2.87)
Total *n*-6 PUFA	17.42 (16.70–18.13)
18:2 *n*-6 (LA)	3.11 (2.67–3.55)
20:4 *n*-6 (AA)	14.31 (13.60–15.01)

Results are given as mean differences (%) (CI). EAT, epicardial adipose tissue; PUFA, polyunsaturated fatty acids; ALA, alpha-linoleic acid; EPA, eicosapentaenoic acid; DHA, docosahexaenoic acid; LA, linoleic acid; AA, arachidonic acid.
